# A pangenome-guided manually curated library of transposable elements for *Zymoseptoria tritici*

**DOI:** 10.1186/s13104-023-06613-7

**Published:** 2023-11-16

**Authors:** Tobias Baril, Daniel Croll

**Affiliations:** Laboratory of Evolutionary Genetics, Institute of Biology, University of Neuchatel, Rue -Argand 11, 2000 Neuchatel, Switzerland

**Keywords:** Transposable Elements, Zymoseptoria tritici, Transposons, TE library, Manual curation, TE consensus

## Abstract

**Objectives:**

High-quality species-specific transposable element (TE) libraries are required for studies to elucidate the evolutionary dynamics of TEs and gain an understanding of their impacts on host genomes. Such high-quality TE resources are severely lacking for species in the fungal kingdom. To facilitate future studies on the putative role of TEs in rapid adaptation observed in the fungal wheat pathogen *Zymoseptoria tritici*, we produced a manually curated TE library. This was generated by detecting TEs in 19 reference genome assemblies representing the global diversity of the species supplemented by multiple sister species genomes. Improvements over previous TE libraries have been made on TE boundary resolution, detection of ORFs, TE domains, terminal inverted repeats, and class-specific motifs.

**Data description:**

A TE consensus library for *Z. tritici* formatted for use with RepeatMasker. This data is relevant to other researchers investigating TE-host evolutionary dynamics in *Z. tritici* or who are interested in comparative studies of the fungal kingdom*.* Further, this TE library can be used to improve gene annotation. Finally, this TE library increases the number of manually curated TE datasets, providing resources to further our understanding of TE diversity.

## Objective

Transposable elements (TEs) are autonomous DNA sequences that can move within the genome. TEs have been implicated in host genome evolution through processes including chromosomal rearrangements, exon shuffling, and donation of coding sequences [1–4]. TEs are highly diverse among eukaryotes and current levels of sampling are insufficient to gain a deep understanding of the evolutionary dynamics of TEs. Compounding this, databases are inundated with putative TE sequences, however only a small fraction of these are curated. For example, in Dfam release 3.7, only 19,730 families (0.57%) are curated out of a total 3,437,876 families [5, 6].

To facilitate evolutionary studies, species-specific TE libraries are needed as TE content can vary significantly, even within a single genus [7]. Thus, TE libraries for even closely related species are not sufficient to accurately characterize the TE content of a genome. Further, TE resources for Fungi are lacking and this impedes studies focusing on genome evolution in this extremely diverse kingdom. *Zymoseptoria tritici* is a fungal wheat pathogen with extensive standing genetic variation within and among distinct populations across the globe [8, 9]. Consequently, parallel evolution across geographic regions has enabled the pathogen to rapidly overcome host resistance and tolerate fungicides in extremely short timeframes [10]. This rapid adaptation, combined with variable TE loads within and among populations [8], makes *Z. tritici* a fascinating system for investigations into TE-host evolutionary dynamics.

To enable studies on evolutionary dynamics of TEs in *Z. tritici*, we present an improved manually curated TE consensus library constructed from a 19-genome reference panel and the sister species of *Z. tritici*. Improvements have been made on TE boundary resolution, detection of ORFs, TE domains, terminal inverted repeats, and class-specific motifs. We have also reduced redundancy in the library.

### Data description

Putative TE consensus sequences were first obtained by annotating all 23 reference-quality genome assemblies [11–25] with Earl Grey (v3.0; https://github.com/TobyBaril/EarlGrey) [26, 27]. Consensus sequences generated from each reference genome were clustered using CD-Hit-Est (v4.8.1) [28, 29] to group sequences with 90% similarity across 80% of the longer sequence length to reduce redundancy whilst preventing the collapsing of chimeric sequences. Each consensus sequence was then subject to manual curation as described by Goubert et al. (2022) [30]. Briefly, genomic copies of each TE were obtained using a “BLAST, Extract, Align, Trim” process to recover genomic copies from each of the 23 reference genome assemblies with 1000 flanking bases at either end [30, 31]. For families with > 100 BLASTN hits, the 25 longest hits were selected, along with 75 random hits. Multiple alignments were generated for each putative TE family using MAFFT (v7.505) with the –auto flag [32]. Columns composed of >  = 80% gaps were removed with T-COFFEE (v13.45.0.4846264) [33]. Subsequently, all sequence alignments were manually curated to define TE boundaries and remove regions of low conservation and rare insertions. Following manual curation, new majority-rule consensus sequences were generated with EMBOSS (v6.6.0.0) cons [34]. TE-Aid (https://github.com/clemgoub/TE-Aid/) was used to aid visual inspection and to identify diagnostic features for classification of extended consensus sequences. Following this, TIRs were recorded (if present), and nhmmscan (HMMER v3.3.2) [35] was used to identify homology to known curated elements in Dfam (v3.7). Combining this information, each TE consensus sequence was manually classified using available information following the naming convention ‘ > ZymTri_2023_family_[n]#[Classification]/[Family]’ for compatibility with RepeatMasker [36]. Consensus sequences classified with low confidence have a ‘?’ added to the name, as well as the string ‘_LowConf’. To reduce redundancy in the final TE library, sequences were clustered to the family-level using the ‘80–80-80 rule’ (*i.e.* ≥ 80% identity, ≥ 80% length, ≥ 80 bp) [30, 37] implemented in CD-hit-est. The representative sequence for each cluster was manually selected to retrieve the sequence with the highest classification confidence, also defined as the ‘most intact consensus’. Chimeric sequences erroneously clustered were manually separated to retain sequences for the chimeric TE and the individual elements that generated the chimera.

In total, we curated 331 distinct consensus sequences for the final TE library (Table 1). Of these, 199 could be confidently classified and 105 consensus sequences remain putative TEs labelled in the library as ‘Unclassified’. The 27 remaining TE consensus sequences are classified with low confidence. TE families from all major classifications are present: 92 DNA transposons, 22 long interspersed nuclear elements (LINEs), 65 long terminal repeat retrotransposons (LTRs), 31 miniature inverted terminal repeat elements (MITEs), 11 rolling circles (also known as helitrons), 1 short interspersed nuclear element (SINE), 1 terminal-repeat retrotransposon in miniature (TRIM), and 105 unclassified elements. The TE consensus library in FASTA format is supplied in data set 1 (Table 1) and a Tar archive containing the annotation of the 19 reference genomes in GFF format is supplied in data set 2 (Table 1).

**Table 1:** Overview of data files/data sets

Label	Name of data file/data set	File types (file extension)	Data repository and identifier (DOI or accession number)
Data set 1	ZymTri_2023.manCurTE.v1_0.fasta	FASTA (.fasta)	Zenodo (https://doi.org/10.5281/zenodo.8379981) [38]
Data set 2	*.TE_2023.gff	General Feature Format (GFF)	Zenodo (https://doi.org/10.5281/zenodo.8390461) [39]

## Limitations

Whilst we made use of large public databases and an extensive set of genomes, 105 TE consensus remain unclassified and an additional 27 are classified with low confidence. Further manual curation efforts following sampling of more genome assemblies might aid in the classification of these by providing additional diagnostic features.

Limited knowledge on the diversity of TEs across the fungal kingdom may have impacted our ability to classify sequences to family-level. We anticipate this limitation will become less significant as genomic sampling and TE curation across the kingdom expands.

The integration of nearly two dozen reference-quality genomes significantly improved our ability to identify even low-copy TEs in the species. However, the dynamic nature of TE activation and repression within the species [8, 40] poses a significant challenge to capture the full TE content of the species. Hence, some recently reactivated TEs or very low-copy number TEs might have evaded detection, and so be missing from the final library.

## Data Availability

The manually-curated TE library for *Zymoseptoria tritici* can be freely accessed on Zenodo at https://doi.org/10.5281/zenodo.8379981 [38]. The TE annotations for the 19 *Zymoseptoria tritici* reference genomes can be freely accessed on Zenodo at https://doi.org/10.5281/zenodo.8390461 [39].

## Abbreviations

TEs: Transposable elements
LINE: Long Interspersed Nuclear Element
LTR: Long Terminal Repeat
MITE: Miniature Inverted Terminal repeat Element
SINE: Short Interspersed Nuclear Element
TRIM: Terminal Repeat In Miniature
